# Editorial: Autophagy in endocrine-metabolic diseases associated with aging: Volume II

**DOI:** 10.3389/fendo.2024.1439492

**Published:** 2024-06-17

**Authors:** Maria Ines Vaccaro, Vincenzo De Tata, Claudio Daniel Gonzalez

**Affiliations:** ^1^ Institute of Biochemistry and Molecular Medicine Prof Alberto Boveris, University of Buenos Aires, National Council for Scientific and Technical Research (CONICET), Buenos Aires, Argentina; ^2^ Department of Translational Research and New Technologies in Medicine and Surgery, University of Pisa, Pisa, Italy; ^3^ Center for Medical Education and Clinic Research (CEMIC) University Institute, Buenos Aires, Argentina

**Keywords:** autophagy, metabolic diseases, senescence, exercise, insulin, retinopathy, SIRT1, aging

Macroautophagy is a cellular process that sequesters senescent or damaged organelles and proteins in autophagosomes for recycling their products. Autophagy is also involved in the removal of cells that have undergone classical apoptosis. Hence, autophagy can be generally considered as a protector of cells against various stressors and a cellular response to routine wear-and-tear. Paradoxically, autophagy may also be related to a form of non-apoptotic program cell death. Thus, autophagy can either protect cells or promote cell death, depending on the cellular and environmental context. Changes in autophagy are early events within the course of many degenerative diseases frequently associated with aging. Some of these modifications clearly precede the clinical diagnosis of these diseases and might mark the evolution from pre-clinical to overt diseases. Endocrine-metabolic diseases like type 2 diabetes, thyroid dysfunctions associated with the aging, non-alcoholic fatty liver disease and many forms of endocrine cancer are closely linked with disturbances into the autophagy flow. Even endothelial alterations related to these metabolic disturbances, including atherogenic processes, are associated to these modifications. Chronic complications of metabolic diseases are also linked to alterations in autophagy rates and flux. Sarcopenia and neurological age- associated degenerative diseases, such as Alzheimer disease, are pathophysiologically related to different forms of cell dysfunction linked to macro autophagy impairment. Imbalances in the antioxidant capacity within the cell result in oxidative stress-mediated injury. Selective autophagy mechanisms, as well as vesicular secretion and pathways related to senescence are relevant topics involved in the pathophysiology of these complex diseases.

Ageing and age-related degenerative diseases are associated with relevant alterations in autophagy. Some metabolic factors linked with age could connect aging with these dysfunctions in autophagy. For example, insulin resistance tends to increase with age. This insulin resistance is often associated with hyperinsulinemia. Hyperinsulinemia and insulin resistance are observed in patients with obesity, prediabetes, and diabetes (especially in the early stages of disease progression), and they have been linked to alterations in bone and muscle metabolism, cardiovascular disease, and even cognitive impairments, among others. Some studies have focused on their association with malignant lesions in various organs, including the breast, colon, among others.

Although insulin plays a crucial role in the growth and maintenance of complex organisms, numerous findings indicate that heightened insulin levels contribute to age-related ailments. The anabolic nature of insulin leads to cellular stress due to heightened biosynthetic activity and decreased clearance of damaged cellular components. Increased insulin levels, often associated with insulin resistance, accelerate aging through various mechanisms. In instances of hyperinsulinemia and insulin resistance, heightened mTOR signaling and suppression of FOXOs may partially explain the inhibition or disruption of autophagy, thereby contributing to aging and certain autophagy-related age-associated diseases and complications. Kolb et al. delve into these mechanisms and their potential repercussions by examining the intricate interplay between insulin and aging, including their impact on autophagy regulation.

Diabetic retinopathy (DR) is a frequent complication of diabetes mellitus. DR is a leading cause of blindness in the developing world. Moreover, retinopathy is also observed in a proportion of patients at pre-diabetic stages. Hyperglycemia is clearly associated with the initiation and progression of this complication through several mechanisms involving glucose associated oxidative stress, osmotic damage, them generation of AGE (Advanced Glycated End-products) among other factors and mechanisms (1). Diabetes is frequently associated with hypertension and dyslipidemia. Both comorbidities increase the risk for DR. Diabetes-induced damage to the retina manifests in two distinct subtypes. The initial phase, termed no proliferative diabetic retinopathy (NPDR), is characterized by the formation of exudates due to the leakage of blood vessels within the retina. Typically asymptomatic in its early stages, NPDR seldom results in noticeable vision impairment. Conversely, proliferative diabetic retinopathy (PDR) denotes a more severe progression of the condition. PDR arises when fragile new blood vessels develop atop the retina, posing the risk of hemorrhaging into the vitreous humor. DR may also lead to diabetic maculopathy with new blood vessels developed on the macular section of the retina. It is associated with leaking or blocking of circulation. Without intervention, PDR can culminate in complete blindness (2). Progressive deterioration of retinal structure and physiology are accompanied with clear signs of retinal cell senescence. Most retinal cell types are affected by hyperglycemia. Retinal ganglion cells are projection neurons that make a critical connection between photoreceptors and the brain. Retinal ganglion cells loss plays a significant role in the pathophysiology of DR and its clinical features. To explore the expression of some genes of interest in Retinal ganglion cells, after a bioinformatic exploration, Peng et al. constructed an experimental model using R28 cells exposed to high glucose concentration with the intention to reproduce (up to a certain limit) some of the DR metabolic microenvironment conditions. R28 retinal precursor cell line is a well know lineage stablished more than two decades ago. As a result of their research, these authors suggest that some potential mechanisms associated with autophagy and senescence are upregulated under high glucose concentrations. Some autophagy and senescence-related genes, such as TP53 and CDKN2A are upregulated retinal cells involved in DR. They also suggest that autophagy mechanisms are challenged under high glucose concentrations, limiting its potentially protective effects. Considering all the potential, well disclosed limitations associated to this model, this study proposes an interesting autophagy associated research line that deserves for further investigation. The exploration of these insufficiencies in autophagy-mediated neuroprotection may provide ground for further therapeutic developments.

Ferritinophagy has been proposed as a selective autophagic degradation of ferritin, resulting in cytosolic iron overload under its ferrous configuration (Fe2+). While hereditary ferritinopathies are rare forms of genetic alterations of iron homeostasis (most of them explained by mutations in ferritin chain genes), some acquired abnormalities in ferritin metabolism seem to be part of the pathophysiology of certain more frequent diseases. Under certain conditions, exaggerated ferritinophagy may result in prooxidative intracellular conditions, including lipid and protein oxidation and reactive oxygen species generation associated with iron overload. These conditions may drive to severe cell damage and death. Inhibition of ferritinophagy is followed by a reduction of ferroptosis in myocardial ischemia/reperfusion models. Ferritinophagy dysregulation is also associated with some forms of retinal disease, including retinopathy. Increased ferroptosis and ferritinophagy was also observed in other diabetes complications. Yu et al. identified and proposed specific autophagy- associated molecules (particularly BECN1, HERC2, ATG7, and BCAT2) as potential biomarkers for diabetic retinopathy (DR). These molecules may modulate ferritinophagy and the immune microenvironment, thus influencing the onset and progression of the condition. This represents another intriguing avenue of research to pursue in order to gain a deeper understanding of the pathophysiology of DR.

Senile osteoporosis and its consequences - bone fragility and fracture susceptibility - contribute significantly to the development of frailty in the elderly. In their article, Zhu et al. evaluated the possibility to prevent or delay the onset of this age-associated impairment of bone metabolism in mice by physical exercise. They demonstrated that in aged (12-mo-old) mice, reductions in bone mass and bone mineral density are associated with decreased autophagic activity with respect to young (5-mo-old) mice. Interestingly, 8 weeks of treadmill running exercise upregulated autophagy in bone cells and at the same time improved both bone mass and bone mineral density in aging mice. The decreased autophagy in old mice was correlated with a parallel reduction in Sirtuin 1 (SIRT1) mRNA expression, whereas physical exercise was able to restore its expression to the levels of young mice. SIRT1 overexpression in exercise-trained old mice was correlated with the increase of several markers of autophagy activation. Recent studies have shown that Sirt 1 can contribute to the regulation of the activities of bone tissue. SIRT1 can promote the osteogenic differentiation of human bone marrow mesenchymal stem cells (BMSCs) and counteract the osteoclast-mediated bone resorption (3, 4). SIRT1 has been shown to play a key role in the regulation of autophagy (5). Zhu et al. demonstrated that *in vitro* mechanical stretching increases the expression of osteogenic genes in isolated BMSCs, whereas SIRT1 knockdown reduces this effect. In conclusion, the results of Zhu et al. indicate that physical exercise, probably by the activation of the SIRT1-autophagy axis, can have a positive effect in the prevention of age-related changes in bone tissue.

This second volume of our Research Topic has examined the intricate interplay between insulin, aging and autophagy; has explored the autophagy-mediated neuroprotection. Also has identified some autophagy related molecules that may modulate ferritinophagy; and has presented evidence supporting that physical exercise can have a positive effect in the prevention of age-related changes in bone tissue. Further questions may arise as natural consequence: Is autophagy a target for therapeutic intervention in these degenerative diseases? If affirmative, what is needed to do? What are the optimal autophagy rates and/or flux to be obtained at the critical tissues? Aligned with the answers to these and other related questions might help to the development of new therapies to take advantage of the potential cytoprotective effect of autophagy in chronic degenerative diseases as a potentially promising avenue of investigation.

## Author contributions

MV: Writing – review & editing, Writing – original draft. VT: Writing – original draft. CG: Writing – review & editing, Writing – original draft.
